# Applying the Nonadoption, Abandonment, Scale-Up, Spread, and Sustainability (NASSS) Framework to Adapt the CHAMP App for Pediatric Feeding Tube Weaning: Application and Case Report

**DOI:** 10.2196/67398

**Published:** 2025-06-16

**Authors:** Dana M Bakula, Alexandra Zax, Sarah Edwards, Kristina Nash, April Escobar, Rachel Graham, Amy Ricketts, Ryan Thompson, Sarah Bullard, Julianne Brogren, Leah Shimmens, Lori A Erickson

**Affiliations:** 1 Department of Pediatrics, Children's Mercy Kansas City, 2401 Gillham Rd, Kansas City, MO, 64108, United States, 1 (816) 302-3069; 2 School of Medicine, University of Missouri-Kansas City, Kansas City, MO, United States; 3 Center for Children’s Healthy Lifestyles and Nutrition, Kansas City, MO, United States; 4 University of Kansas, Lawrence, KS, United States; 5 Remote Health Solutions, Children’s Mercy Hospital, Kansas City, MO, United States; 6 School of Nursing, University of Missouri-Kansas City, Kansas City, MO, United States

**Keywords:** gastric feeding tubes, mobile applications, interdisciplinary communication, feeding and eating disorders of childhood, case report

## Abstract

**Background:**

Evidence-based tube feeding (TF) weaning involves reducing the volume of tube feeds to induce hunger, with interdisciplinary monitoring to allow for proactive medical, nutritional, and behavioral intervention as needed. This can be done outpatient; however, the current standard of care requires a high degree of medical monitoring and care coordination, which can be challenging to implement. The CHAMP App is a mobile app designed for remote patient monitoring of children born with congenital heart conditions who are at high risk for medical morbidity and mortality. The CHAMP App remote patient monitoring program would be ideally suited to improve medical monitoring and care coordination.

**Objective:**

This study aims to determine the feasibility of adapting the CHAMP App for children ready to wean from TF.

**Methods:**

We used the Non-adoption, Abandonment, Scale-up, Spread, and Sustainability (NASSS) framework as a formative tool and conducted a case study beta test.

**Results:**

The level of complexity for the digital innovation’s adaptation supported a high likelihood of success for the TF population. Most issues were simple, such as expanding the types of data that could be entered into the app, and some were more complicated, for instance, training all relevant staff to use and maintain the technology. The case study beta test was conducted with “Greyson”, a 10-month old child weaning from TF (name changed for confidentiality). Once a week, the team reviewed the parent-entered data and communicated with Greyson’s parents, recommending a 25% reduction in tube feeding each week. With the CHAMP App facilitating 2-way communication between the family and the team, Greyson successfully transitioned from receiving 30% of his feeds orally and 70% via tube feeding to 100% oral feedings over the course of 1 month in a home setting.

**Conclusions:**

The CHAMP App is likely to be incredibly valuable in TF weaning. The NASSS framework helped identify key considerations for adapting the CHAMP App for TF weaning at a Midwestern children’s hospital. Interviews with the health care team highlighted issues like data entry expansion and staff training. The framework confirmed TF weaning as a suitable application with no major barriers. The CHAMP App successfully supported a test patient, Greyson, in weaning from his feeding tube. It may improve access, communication efficiency, and satisfaction among families and health care teams while reducing costs and enhancing safety monitoring. The app could also make TF weaning more accessible to families with lower health literacy.

## Introduction

Enteral tube feeding (TF) is a lifesaving measure, but the prolonged use of TFs is taxing on the child’s and family’s well-being [1]. TF requires complicated medical devices, access to formulas or blended foods that must be prepared and stored in specific ways, and 24/7 management, resulting in a high burden on the family of TF children and limitations to the TF child’s independence [1-3]. Caregivers report that TF comes with the “loss of a normal life,” negative psychological impacts, practical challenges, and the need to become the “expert” [1]. Thus, the goal is to wean from TF once a child is able to achieve weight stabilization and improved safety in eating by mouth [2,4].

Evidence-based TF weaning involves gradually reducing the volume of tube feeds to induce hunger under the guidance of a multidisciplinary team so that behavioral, medical, and nutrition interventions can be implemented as needed to support tube weaning [5]. It is particularly critical to prevent dehydration and excessive weight loss during TF weaning, as these can be dangerous and lead to other health problems [6]. With the appropriate monitoring and interdisciplinary support, outpatient TF weaning is incredibly effective (67%‐69% successful) [5]. However, the current standard of care requires a high degree of care coordination and communication between families and the medical team. This can be challenging to implement using traditional models of outpatient care that rely on parent-initiated contact between outpatient clinic visits [5,6].

Mobile health (mHealth) apps and software platforms that allow for remote home monitoring have become common strategies to monitor weight and growth in other pediatric specialties, such as cardiology [7]. The CHAMP App is a proprietary software platform developed in 2014, with key stakeholder input from parents and health care providers, for asynchronous data transfer by parents at home to their specialty health care teams, starting with the pediatric cardiac population [8]. Through this model, pediatric cardiology researchers have reported improved communication, pediatric survival through staged surgeries, growth, and fewer intensive care days [9]. We hypothesize that expansion of the CHAMP App adaptation to pediatric TF weaning would be an optimal way to improve the TF weaning process.

Our study objective is to describe the use of the Non-adoption, Abandonment, Scale-up, Spread, and Sustainability (NASSS) framework as a formative tool in identifying the potential fit between the CHAMP App and TF weaning. The NASSS framework is an implementation science framework aimed at predicting and evaluating the success of mHealth interventions [10]. We will also describe a case example of CHAMP for TF weaning, which we have labeled “CHAMP for the Feeder.”

## Method

### Study Design

We used the NASSS framework to determine the feasibility of adapting the CHAMP App for children ready to wean from TF. The NASSS framework is organized by seven domains: (1) condition; (2) technology; (3) value proposition; (4) adopter system (5) organization; (6) broader context; and (7) embedding [10,11]. Each domain has a set of questions to answer and review in a mixed-methods approach from primary or secondary data sources, expert knowledge, and interviews with key stakeholders [10]. Each domain is categorized by (1) simple: straightforward, predictable, and have few components, (2) complicated: multiple interacting components or issues, or (3) complex: dynamic, unpredictable, and prove difficult to disaggregate into constituent components [11].

### Setting

The organization is a Midwest, free-standing tertiary children’s hospital with multiple inpatient and outpatient locations. The hospital has 390 inpatient beds over two locations, and multiple ambulatory care clinics across the region. Nearly 9000 employees care for children before birth to 18+, from all 50 states, 49.6% medicaid payor source, and over 14 countries internationally [12].

#### Interdisciplinary Feeding and Swallowing Program

The organization’s Interdisciplinary Feeding and Swallowing Program (IFSP) is a specialized outpatient clinical team within the Gastroenterology Department [13]. The IFSP specializes in providing integrated care, from multiple specialists in one location for families of children with medical complexities and feeding difficulties. The program has a team of more than 30 specialists including gastroenterology physicians, nurse practitioners, nurse coordinators, speech-language pathologists, occupational therapists, dietitians, psychologists, and social workers [13]. This program conducts more than 2000 patient visits per year, and each year sees over 300 new patients.

#### Remote Health Solutions Team

The Remote Health Solutions (RHS) team is a department within the Division of Strategy, Innovation, and Partnerships. Their focus is on digital health models that can apply a personalized, proactive approach to remote asynchronous monitoring. This team includes nurse researchers, research nurses, clinical research coordinators, and program coordinators.

### Description of the NASSS Framework Process

The NASSS framework is applied through planning and feasibility stages to guide conversations, and identify implementation barriers and facilitators. Semi-structured meetings were completed to evaluate feedback reflexively to increase answers to the questions from the 7 domains(Textbox 1). We met with stakeholders with varying expertise inside the organization. Interviews were conducted as virtually in small groups. Stakeholders included leaders with knowledge of remote health and mHealth platforms, care of children with tube feedings, pediatric providers, administration, strategy, innovation, ambulatory nursing, information systems, software development, patient and family partners, and research. Administrative data were used to evaluate feasibility and planning via patient volumes and incidence.

Textbox 1. **Non-adoption, Abandonment, Scale-up, Spread, and Sustainability (NASSS) framework for planning and feasibility management by the research leadership team.**

The questions were used to guide semi-structured interviews, and the summarized themes were then coded to reflect simple, complicated, or complex issues.

**Condition or Illness**

• What is the nature of the condition or illness?

• What are the relevant sociocultural factors and co-morbidities?

**Technology**

• What are the key features of the technology?

• What kind of knowledge does the technology bring into play?

• What knowledge or support is required to use the technology?

• What is the technology supply model?

**Value Proposition**

• What is the developer’s business case for the technology (supply-side value)?

• What is its desirability, efficacy, safety, and cost-effectiveness (demand-side value)?

**Adopter System**

• What changes in staff roles, practices, and identities are implied?

• What is expected of the patient (and or immediate caregiver)- and is this achievable by and acceptable to them?

• What is assumed about the extended network of lay caregivers?

**Organization**

• What is the organization’s capacity to innovate?

• How ready is the organization for this technology-supported change?

• How easy will the adoption and funding decision be?

• What changes will be needed in team interactions and routines?

• What work is involved in implementation and who will do it?

**Wider Context**

What is the political, economic, regulatory, professional (eg medicolegal), and sociocultural context for program rollout?

**Embedding and Adaptation Over Time**

How much scope is there for adapting and coevolving the technology and the service over time?

### Analyses

Feedback from leaders, field notes from interviews, and administrative data were compiled. Interview themes were collated and key points were identified based on core components of the NASSS framework. Project leaders from the IFSP and RHS teams from the Gastroenterology and Strategy, Innovation, and Partnerships departments collaboratively reviewed participant responses via typed transcripts and meeting notes and conducted an inductive thematic analysis, guided by Morgan and Krueger methodology, to identify themes within the responses [14]. The project leaders met as a group to reach a consensus on theme content areas and to develop the final wording of themes. Questions and notes for each domain were answered by the project leaders and confirmed with division and executive leadership at the organization for agreement on the planned program’s complexity. Generative artificial intelligence (AI) models were not used for any portion of the research project or writing of the study findings.

### Ethical Considerations

Interviews with key stakeholders as part of the NASSS formative research process were deemed not to be human participants research and therefore did not involve consent procedures. Stakeholders were recruited to participate in semi-structured meetings through email and phone communication starting in late 2020. For the beta case testing of the CHAMP App for tube weaning, we received institutional review board (IRB) approval (Study #00001436) before study activities commencing in 2021. The parent was provided with all relevant study information and educated on the study procedures by a trained study staff member and given the option to opt in or out of the study. Upon agreeing to participate, the parent signed the IRB-approved informed consent to allow their child’s data to be used in a clinical repository. In addition, the parent agreed to a secondary analysis case study report centered on their experience using the CHAMP App for TF weaning, as well as consented to share deidentified study data. All data presented here has been deidentified. There was no compensation provided to human participants research participants in this study.

## Results

### Domain 1: Condition

In line with the first domain of the NASSS framework, we began by considering the nature of the condition (ie, weaning from TF) to determine whether the CHAMP App might be appropriate for children ready for TF weaning. We determined that TF weaning was an appropriate population for the CHAMP App, and that the level of complexity needed to use the app to this population was simple based on the following key points: (1) The population who receive TF is well characterized and understood (26.99‐44.25 per 1000 children <5 y of age require TF [15,16]). (2) Children with other medical conditions, or who are born prematurely, are more likely to need TF to support their health and development, and therefore, TF is often well understood by medical teams across most major pediatric tertiary care settings. (3) The TF weaning process requires close medical monitoring, which maps well to the capabilities of the CHAMP App. Specifically, TF weaning involves slowly reducing the amount of nutrition delivered via TF so that the child can eat more by mouth. Medical monitoring ensures that the child maintains age-appropriate growth and does not experience adverse consequences such as dehydration or malnutrition. (4) TF weaning in outpatient and home settings is ideal, with interdisciplinary oversight from physicians or nurse practitioners, nurses, psychologists, dieticians, and feeding therapists (speech and occupational therapists) [2]. This approach aligns well with the original use of the CHAMP App for use in the home setting.

### Domain 2: Technology

**Figure 1.**
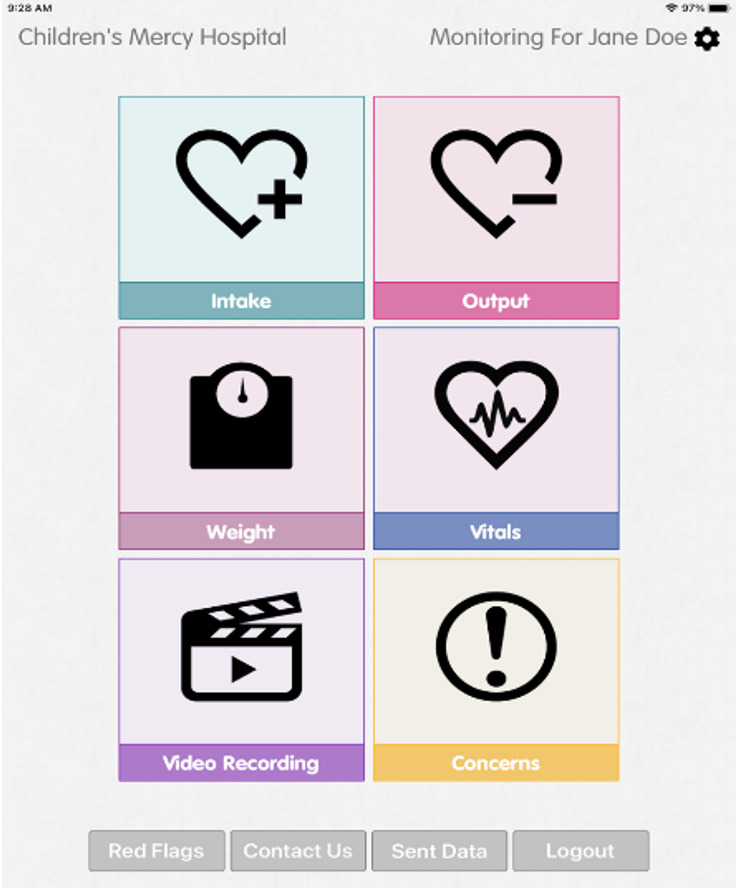
CHAMP App user interface screenshot.

**Figure 2.**
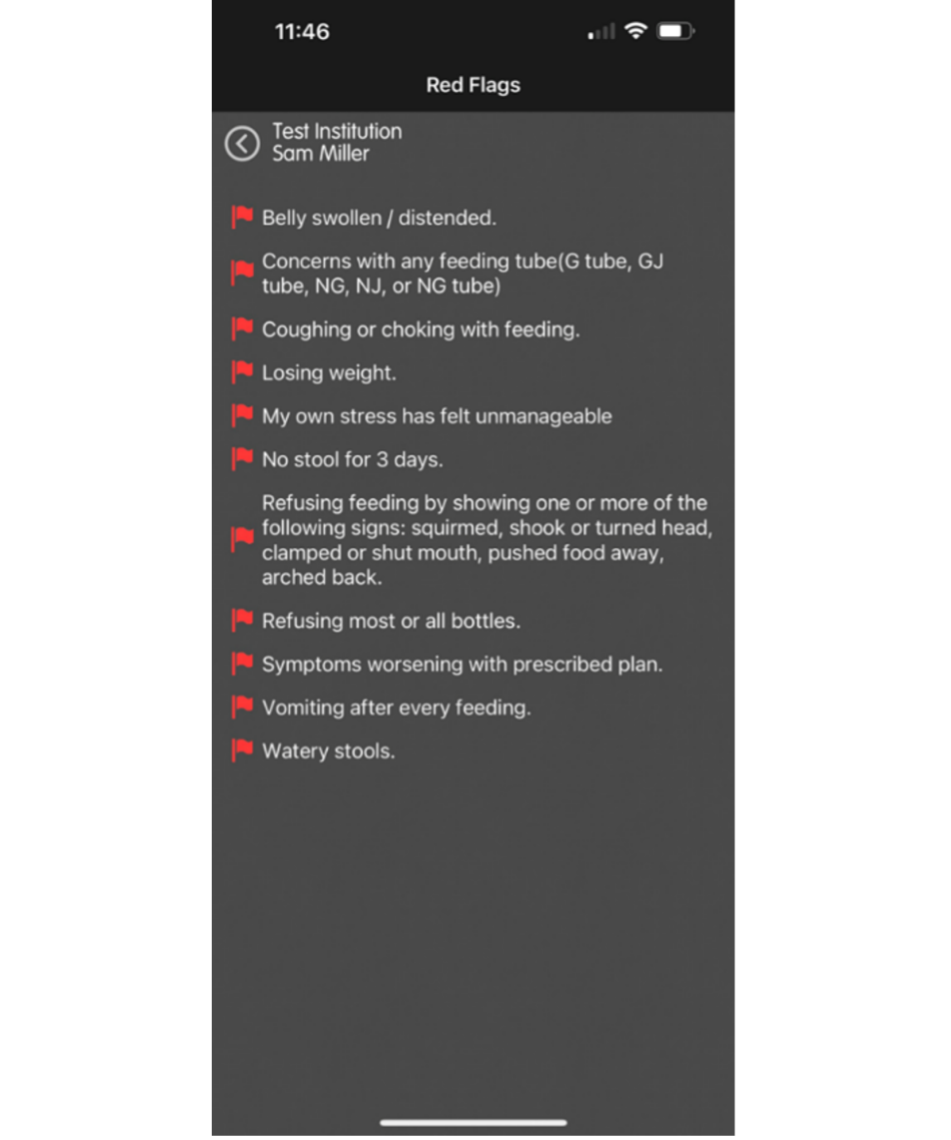
CHAMP App red flags function screenshot.

**Figure 3.**
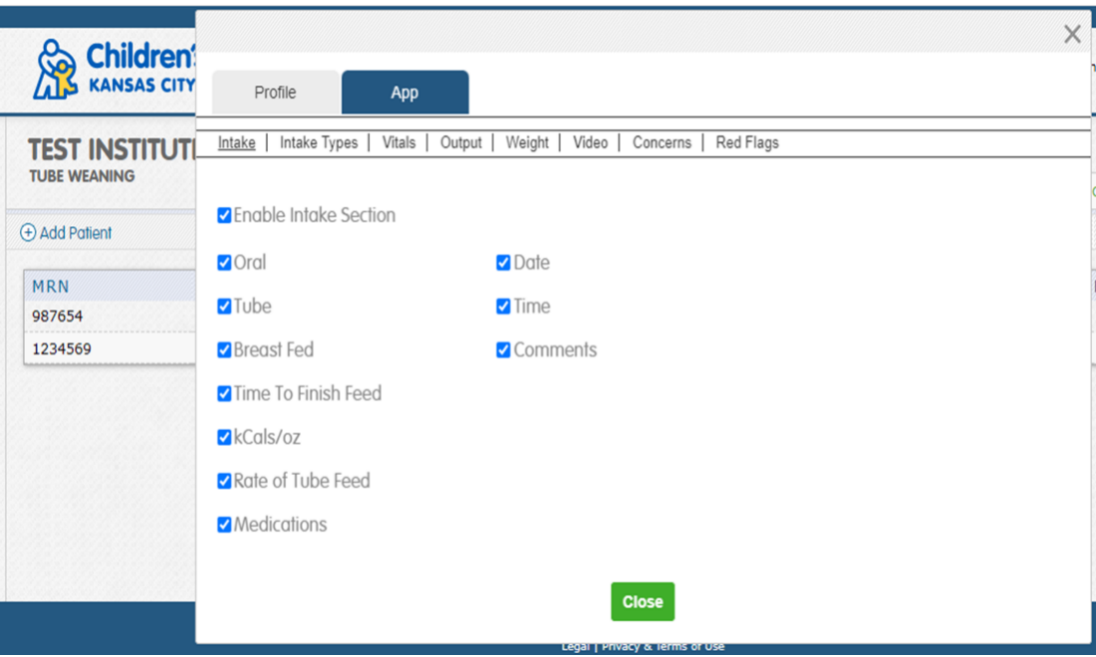
CHAMP App provider dashboard screenshot.

We determined that TF weaning was an appropriate fit for the CHAMP App technology, and that the level of complexity needed to adapt the technology to this population was simple based on the following key points: (1) First, is the successful implementation and dissemination of the CHAMP App in cardiology. The CHAMP App was developed for data transfer with a pediatric cardiac population and provides families and the clinical team with a new model of care leveraged by a mobile app integrated with a software platform [17]. Data options include feeding intake, output, vital signs, weights, videos, and concerns (Figure 1). Since 2014, the CHAMP App in the pediatric cardiac population has been used in over 1000 patients, 28 states, and across 12 pediatric hospitals and is available in 11 languages for families to use on their mobile phones in research formats (NCT 15030113CMH). Nurses review the data intermittently and communicate with families using proactive data review in place of standard parent-driven communication in a reactive format in ambulatory clinics. Data is transferred to a health care team web portal which allows for early identification and feeding and growth intervention [9]. The CHAMP App is free-standing and dependable as an app with inclusion of more than 1100 patients across 12 pediatric hospitals in a pediatric research registry. The ability for proactive data review has led to reports of improved survival outcomes, no instances of malnutrition, and fewer episodes of morbidity with less intensive care days [9]. (2) The CHAMP App has easy access in mobile version and web-based health care team dashboard, simple sets of instructions, verified training methods, and manuals developed in multiple languages for smooth implementation. This would allow easy usability of the pp for new purposes. (3) The cloud-based technology supply model for CHAMP App is customizable and easy to adapt for new purposes. The app is designed to be generic with “plug and play” features on both the parent-facing and health care team dashboards and reports. To tailor the CHAMP App for the TF weaning population, minimal adjustments to the mHealth technology were needed, such as the ability to report additional feeding measures (Figure 2), red flags for parents of children being TF weaned (Figure 3).

### Domain 3: Value Proposition

During the value proposition phase, we evaluated the developers’ business case for the supply-side and demand-side values of the technology and determined that the level of complexity needed to supply use of the CHAMP App for children undergoing TF weaning across multiple clinics was simple based on the following key points: (1) since the CHAMP App is a proprietary software platform developed by the organization, the hospital derives benefits from the use of the app and dissemination of the app. The return on this investment with improved patient outcomes reduced health care costs related to maintaining TF technology, family experience, and innovation in remote home monitoring internally and externally far outweighed the cost of expansion with staff time and financial instances. (2) The demand-side value is high because the app is desirable, efficacious, safe, and cost-effective. CHAMP App had a good adherence rate with nearly 75% for families of children with cardiac disease [17]. Over 90% of caregivers offered, consent to use the CHAMP App over paper documentation by parents [8]. (3) This project will fill an existing demand at our institution. The IFSP team at our institution has a high demand for appointments for new and follow-up patients and more children are being discharged each year from the organization with feeding needs. From administrative data, approximately 200 new children under the age of 5 years have gastrostomy tubes across all outpatient clinics per year. The incidence and value of an innovative support model for children with TF were displayed.

### Domain 4: Adopter System

In line with the adopter system phase of evaluation, we considered the current standard of care for TF weaning and determined that the level of complexity to adapt the app within the adopter system was complicated based on the following key points, (1) when evaluating critical changes in staff roles, practices, and identities, it was determined that a front-line nutrition team member was necessary for successful adoption of the CHAMP App. This team member would need to be readily available to review data, communicate with families during the 4‐6-week protocol wean, and be responsible for early interventions when weaning questions and problems arose. Further, the existing nutrition and feeding team staff would need to learn how to use CHAMP health care web portal. This change was informed by the standard of care which involves frequent and systematic communication between the health care team and family to identify readiness with oral intake, receive support from speech and feeding therapists, and acquire clinical approval from the specialty health care teams. During the tube wean itself, families rely on communication with the health care team for guidance on reduction of calories from the feeding tube over a short period of time, with the intent to increase hunger and subsequently oral intake. In outpatient settings for TF weaning, parents communicate with the health care team at clinic visits and through reactive health care communications initiated by parents to the health care team. In some cases, practices report inpatient admissions for weaning so that patients are guaranteed access to holistic team support; however, the disruption for families and high cost of readmission is limiting [18]. Therefore, a front-line nutrition team member would guarantee that families had support and access to the health care team while also minimizing disruption and cost for families.

It was noted that parent engagement would be key to successful implementation. The organization’s cardiac parent family advisory board had specifically asked for an innovation like this for their children. Given the success of adoption and adherence among parents of the pediatric cardiac group [9,19], it was expected that login to the CHAMP App and the patient portal would also be acceptable for parents of children prepared for TF weaning. (3) Finally, we considered the adoptees’ network of lay caregivers and determined that this was not particularly relevant to the success of the app with this population given that data entry would be asynchronous and would initially follow a research protocol and not an extended care network. Further, larger scale implementation may also increase the use of extended caregivers, such as primary care providers, and a report system is built into the web portal for instances when parents require additional support.

To address these complicated issues, we determined that some work would be needed to build a shared vision, engage staff, enact new practices, and monitor the impact of the CHAMP App. TF weaning specific handouts, research protocols, and grant submissions for a project manager nurse were submitted and completed to address existing gaps. Further, weekly meetings continued with the RHS and IFSP teams and included short bursts of projects and long-term planning for implementation.

### Domain 5: The Organization

In line with Domain 5 of the NASSS framework, we considered our organization’s capacity to innovate and determined that the level of complexity for adaptation within said organization was simple*-*complicated based on the following key points: (1) The organization is known for a culture of pediatric innovation with a strong support model for technology innovations. Therefore, the risk involved in customizations of the CHAMP App for TF weaning was deemed appropriate at an organizational level. Notably, the organization has a high tension for change, good innovation-system fit, widespread internal support, and supported the research team’s desire to submit for external funding with cost-sharing support for the endeavor. (2) Within this domain, we also considered factors such as adoption and funding. Accordingly, our organization had sufficient resources, anticipated cost savings, and required no new infrastructure or recurrent costs to adopt the technology for TF weaning. It was noted that new team routines would need to be aligned with established protocols if teams were not already using the weaning protocol.

### Domain 6: The Broader Context

We considered the broader context that would be relevant to the implementation of the CHAMP App for children undergoing TF weaning and determined that the level of complexity for successful adaptation within the broader context was *simple* based on the following key points: (1) Professional and lay stakeholders were generally supportive and remote patient monitoring for weight gain is in place nationally from the Centers for Medicaid and Medicare Services guidelines from 2023 [20]. (2) While sense-making, collective reflection, and adaptive actions across multiple service lines are more complex compared to other areas within this domain, we were confident that collective reflection with IFSP and RHS teams would allow us to appropriately handle critical events and adapt to unforeseen eventualities.

### Domain 7: Embedding and Initial Implementation

In line with the embedding and initial implementation domain of the NASSS framework, we evaluated the likelihood that app use would spread locally and, eventually, regionally. We determined that the level of complexity was *simple-complicated* based on the following key points: (1) ongoing use cases that have emerged for the technology, as well as continued organizational support for its adaptation and implementation. This suggests that app is generalizable outside of pediatric cardiology alone and may provide potential opportunities locally and even beyond our institution. (2) Further, the application may provide benefits across multiple pediatric specialties, as children with a range of medical comorbidities use enteral feeding for short or long-term needs. Indeed, there are over 15 service lines at our institution that wean children from TF. (3) Finally, the CHAMP App as a software platform continues to have collective reflection and support to continue the innovations, maintenance, and growth in areas that can follow the organization’s mission and values specific to innovation for all children.

In sum, the level of complexity for the digital innovation’s success across domains supported a high likelihood of success. The areas of focus would include primarily the adopter system as it was the domain that had the highest need for support for the implementation and change models.

### Beta Testing: Case Study

**Figure 4.**
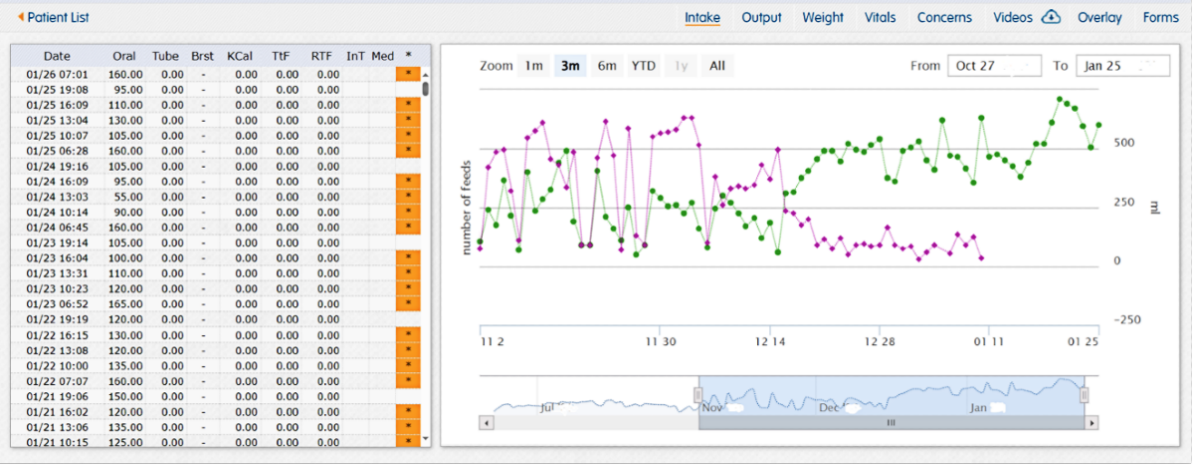
CHAMP tube feeding weaning case study data for “Greyson.” Greyson transitioned from 30% of feeds orally (green dots) and 70% by TF to 100% oral feedings (purple dots).

Finally, we beta-tested this model of CHAMP App for usability with “Greyson,” a 10-month-old child with congenital heart disease and reliance on TF who showed signs of TF weaning readiness including safe oral eating and weight stabilization (name changed for confidentiality). The family, who were familiar with CHAMP from their previous monitoring using the CHAMP App for Greyson’s cardiac monitoring elected to use the CHAMP App for TF weaning. Parents were given a home scale, and entered weights, feeding information, and vital signs multiple times per week, which the IFSP team reviewed in real time. Once weekly after data review, the IFSP team communicated with Greyson’s parents to recommend a TF feed reduction, over a 4-week span (25% volume reduction per wk). With the CHAMP App facilitating 2 way communication between the family and the IFSP team, Greyson transitioned from 30% of feeds orally (green dots in Figure 4) and 70% by TF to 100% oral feedings (purple dots in Figure 4) over one month in the home setting. In addition, his growth was maintained throughout the tube wean with no age-adjusted growth failures when the health care team completed proactive data review and nutritional interventions. The family also reported strong satisfaction with the technology and overall process. Upon 2 year follow up, Greyson still remains on 100% feedings by mouth, demonstrating the promising potential for the expansion of CHAMP in this population.

Following this beta test with Greyson our team identified opportunities to better customize the CHAMP App to optimize it’s use for TF weaning. These changes include allowing for more detailed daily diet record entries, designing TF weaning specific alerts that the family can send to the IFSP team, and modifying the back-end infrastructure to optimally highlight data that is important for TF weaning. These modifications will require collaboration with software teams to implement.

## Discussion

### Principal Findings

The NASSS framework successfully identified critically important considerations for applying the CHAMP App to a new population and conducted a successful pilot of the app for this new purpose with a test participant [10,11]. The NASSS framework was chosen to support our project with a strong background in sustainability and use in more than 1900 research and clinical evaluations of mHealth, including pediatric, remote patient monitoring, and nutrition focused projects [11,21-23]. Using interviews with key multi-disciplinary stakeholders of the health care team involved in TF weaning at a Midwestern children’s hospital, we identified several critical considerations for adaptation of the CHAMP App across domains of condition, technology, value proposition, adopter system, organization, wider context, and embedment/adaptation over time [10,11]. Analysis and thematic understanding of the project were supported by clinical and technology experts to strengthen the data gathered and support the cooperative leadership of the clinical and remote monitoring teams. Executive leadership at the organization, had a strategic position in supporting the project, and provided strong insights into the organizational and broader context of implementation and adaptation internally and externally of the RPM model.

The NASSS-informed interviews allowed our team to recognize that TF weaning was an ideal opportunity for CHAMP App expansion, identified no insurmountable adaptation barriers for TF weaning, and allowed us to develop a roadmap toward successful adaptation and pilot testing. In our initial implementation with a test patient, Greyson, the CHAMP App successfully supported Greyson in weaning from his feeding tube and was acceptable to both the parents and providers involved in weaning. Most barriers were simple, such as expanding the types of data that could be entered into the app, and some were more complicated, for instance, training all relevant staff to use and maintain the technology. Similar to other area pediatric projects, uptake of innovations in an already burdened clinical team takes straightforward and easy to complete training with superior usability of the health care portion of mHealth technologies to encourage adoption [21].

The CHAMP App may have the opportunity to improve access to TF weaning in several ways. The CHAMP App can improve communication efficiency, which may allow for more satisfaction among families and the health care team. As this project moves beyond the research team alone into other areas of the organization, the barriers of teams not yet tube weaning can be addressed through clear steps in the care model to combat drift of sustainability outside the research team [24]. The CHAMP App allows us the opportunity to serve more families than could be served without RPM. Further, the current standard of care for outpatient TF weaning relies heavily on family health literacy and proactive communication with the health care team [2]. The CHAMP App’s RPM system has the potential to be more accessible to families with lower health literacy and allows the health care team to be more proactive in tracking and reaching out to families, which may improve equitable access to TF weaning for all families [25]. In addition, the improved efficiency of TF weaning through RPM may improve outpatient workflow by reducing the number of in-clinic follow-ups needed for children who have successfully weaned from TF through the CHAMP App and, therefore, improve access to the outpatient clinic for new patients.

The CHAMP App may also improve the quality of TF weaning and pediatric nutrition care. Specifically, technology supported models of nutrition care have been identified as a way to allow for more sophisticated safety monitoring as a way to provide high quality nutrition care with appropriate health care team members [23]. As demonstrated by our test case, parent satisfaction is also likely to be high, as this app allows for more seamless support with TF weaning and may help parents to feel more supported and confident in approaching TF weaning. While this was a minimal cost for the test case since they were already used to a technology supported model of care, ongoing costs of implementation in a digital world will need to be followed [23,25]. Further, this app may reduce health care costs, as maintaining TF is expensive [18], and alternatives to outpatient TF weaning are often incredibly expensive as they include inpatient or intensive outpatient treatment which is costly [5].

### Limitations

This study highlights the use of the NASSS framework in identifying a novel opportunity for expansion of a RPM program and is an important first step towards expansion of the CHAMP App. However, several limitations should be taken into consideration when interpreting the findings. While many years of human-centered innovation design and feedback from stakeholders were completed to support this project, we did not conduct a formal qualitative analysis so these findings may be biased by interviewer interpretation. Further, this is a single site study, and involves only a single pilot case. Our team intends to engage in a larger scale implementation of the CHAMP App for TF weaning to better understand the use of this app for TF weaning and to test our indicators of barriers and enablers in scaling beyond the initial championing innovative team with feedback from key stakeholders [22,24].

### Conclusion

The NASSS framework was valuable for identifying important considerations for expanding the CHAMP App to a new population [11]. Health care leaders, innovators, and clinicians may similarly find this framework valuable when planning new mobile technology projects and identifying potential areas of complexity. Based on the outcome of our project, we identified that the CHAMP App is likely to be incredibly valuable and useful in TF weaning, and our team intends to use the information learned in this study to inform the adaptation of the CHAMP App for TF weaning. We will be piloting the CHAMP App for TF weaning in a large cohort of children at a large Midwestern hospital (ClinicalTrials.gov Identifier: NCT06052891).

## Abbreviations

IFSP: Interdisciplinary Feeding and Swallowing Program
IRB: institutional review board
mHealth: mobile health
NASSS: Non-adoption, Abandonment, Scale-up, Spread, and Sustainability
RHS: Remote Health Solutions
TF: tube feeding
